# A general method for elicitation, imputation, and sensitivity analysis for incomplete repeated binary data

**DOI:** 10.1002/sim.8584

**Published:** 2020-07-17

**Authors:** Daniel Tompsett, Stephen Sutton, Shaun R. Seaman, Ian R. White

**Affiliations:** 1 Great Ormond Street Institute of Child Health, UCL, London, UK; 2 Institute of Public Health, University of Cambridge, Cambridge, UK; 3 MRC Biostatistics Unit, University of Cambridge, Cambridge, UK; 4 MRC Clinical Trials Unit, UCL, London, UK

**Keywords:** expert elicitation, MAR, MNAR, multiple imputation, smoking cessation

## Abstract

We develop and demonstrate methods to perform sensitivity analyses to assess sensitivity to plausible departures from missing at random in incomplete repeated binary outcome data. We use multiple imputation in the not at random fully conditional specification framework, which includes one or more sensitivity parameters (SPs) for each incomplete variable. The use of an online elicitation questionnaire is demonstrated to obtain expert opinion on the SPs, and highest prior density regions are used alongside opinion pooling methods to display credible regions for SPs. We demonstrate that substantive conclusions can be far more sensitive to departures from the missing at random assumption (MAR) when control and intervention nonresponders depart from MAR differently, and show that the correlation of arm specific SPs in expert opinion is particularly important. We illustrate these methods on the iQuit in Practice smoking cessation trial, which compared the impact of a tailored text messaging system versus standard care on smoking cessation. We show that conclusions about the effect of intervention on smoking cessation outcomes at 8 week and 6 months are broadly insensitive to departures from MAR, with conclusions significantly affected only when the differences in behavior between the nonresponders in the two trial arms is larger than expert opinion judges to be realistic.

## 1 Introduction

### 1.1 Background

Missing data are common in clinical studies and can lead to biased estimates of treatment effects and misleading conclusions. One solution is to draw imputations based on one or more statistical models for the missing data. These imputation models make one of two assumptions about the missing data: Missing at random (MAR), whereby the probability of being missing depends only on the observed data, and missing not at random (MNAR), where the probability of being missing depends on both the observed and missing data.^1^ Imputation models assuming MAR are often preferred, as they can draw imputations based solely on the observed data. However MAR may be an unrealistic assumption in trial settings, particularly those involving health data, where those who drop out are often expected to respond differently. As MAR and MNAR are untestable assumptions from the available data, it is important to establish how substantive conclusions are affected when the missing data depart from MAR.

MNAR imputation models^2^ include parameters whose pre-specified values are known as sensitivity parameters (SPs), which represent the differences in response between imputed and observed data and govern the extent to which the data deviate from MAR. By repeating the imputation and subsequent analysis over plausible ranges of values for the SPs, one can perform a sensitivity analysis (SA) on substantive conclusions.

Methods for MNAR SAs are well studied, with^2-6^ some examples of work in the area. A popular method of SA is a tipping point analysis^7^ whereby the SPs are increased incrementally until a substantive change in the results (typically the *P*-value) is found. Such methods do not always consider if values of the SP at a tipping point are plausible. Other methods, such as in References 8 and 9 focus on interpreting the SP, and subsequently deriving plausible values or ranges for them. More recent work^10-12^ notes that an SP’s interpretation conditions on the variables included in the imputation models, and suggests they are compared against similar, but easier to understand, SPs or other quantities.

One way to obtain plausible values for SPs is to seek expert advice. This involves asking experts to give prior probability distributions on the relevant outcomes of nonresponders. Expert elicitation has a long history, with^13-16^ some examples of literature in the area, leading to the development of principled methodology such as the “classical mode” approach.^14^ However, experts, sometimes without high statistical knowledge, tend to find constructing defined probability distributions that represent their views a major challenge.

Online elicitation tools have been developed to help experts construct these distributions, and allow for the collection of expert opinion without the need for time-consuming, and often expensive face-to-face meetings. Notable examples include the SHELF framework of References 17 and 18, the MATCH uncertainty elicitation tool of Reference 19, and the R shiny app developed in Reference 9. The latter was designed to be able to quickly elicit information from large numbers of experts, and there is interest in further developing and disseminating such tools for wider use.

Expert elicitation and SA for imputation models involving two or more SPs, such as when separate SPs are defined for nonresponders in each arm, and/or each follow-up time, are very difficult. Additional challenges compared to SA with just one SP include increased computational time and complexity, the construction of plausible regions for SPs in two (or more) dimensions, presenting the SA results, and the consideration that an expert’s opinions about the different SPs may be correlated. A common strategy is to set all SPs to the same value such as in Reference 20, which uses this methodology to find a tipping point. However, this can make strict and unrealistic assumptions about the behavior of nonresponders in different arms, and work on SA methods with multiple varying SPs^9,11,21,22^ is of continued interest.

Elicitation of plausible values can be performed for each SP separately such as in Reference 22, but notes that this enforces the assumption that experts views on each SP are independent. Expert opinion on SPs can be more realistically presented as a joint prior distribution where their beliefs on the correlation between SPs are considered. Constructing such a distribution is, however, a very challenging problem. One such example can be found in Reference 9, which focused on a single, continuous outcome variable with different SPs for missing data in the control and intervention arms. This work included the use of an elicitation app and was built around a fully Bayesian imputation method.

### 1.2 Aims and structure

The main goals of this article are to provide a general method for performing an SA involving two or more SPs. This includes expert elicitation using an online elicitation tool based on that of Reference 9 and graphing credible regions for the SPs using highest density regions (HDR) and linear opinion pooling,^13^ as well as performing and presenting the results of the SA by combining contour plots with the HDRs. We focus on the case of binary data in two arms at two time points, giving us four SPs in total. The methods of the article are applied to the iQuit in Practice (iQiP) smoking cessation trial.^23^ An MNAR SA on a smoking cessation study was conducted in Reference 24 and describes a method with a single SP drawn from a normal distribution derived by simulation, rather than expert elicitation. In this article, we focus on the case of multiple SPs and the elicitation of plausible regions for them imputing using the not at random fully conditional specification (NARFCS) procedure,^10,11^ though our methodology does not rely on a specific imputation method. A template for the elicitation tool of this study is included, as well as step-by-step instructions to perform a similar analysis. This is part of a wider aim to develop a central resource for the general construction of elicitation tools, creation of credible regions, and instructions on how to go about an SA.

The article is structured as follows. The iQiP study is described in Section 2, with the imputation methods of the analysis described in Section 3. In Section 4, we detail the online questionnaire developed for the article and the construction of credible regions. Section 5 details the SA, with results shown and interpreted in Section 6. Section 7 includes instructions to conduct an SA on binary data with two follow-up times and two intervention arms; it also reports the main conclusions of the article.

## 2 The Iquit in Practice Study

The iQuit in Practice study involved 602 eligible participants recruited from 32 general practices in England. The objective was to evaluate a highly tailored, 90-day text messaging system and advice report to aid with smoking cessation, called the iQuit system. Participants were randomized into two arms. The control arm included 303 participants who received usual care, involving an appointment with a smoking cessation advisor (SCA) and general advice for quitting. The intervention arm had 299 participants, who were given usual care plus the iQuit system. Participants agreed with the SCA to set a quit date within two weeks of the appointment. They were followed up at 4 weeks, 8 weeks, and 6 months. The first follow up involved an appointment with an SCA; the other two follow ups involved self-completion questionnaires and were sent by post. The primary outcome of interest was self-reported two-week point prevalence abstinence at 8-week follow-up. There was a nonnegligible amount of missing data. The control arm had missing data on smoking cessation in 32%, 18.2%, and 21.5% of participants at 4 weeks, 8 weeks, and 6 months, respectively. For the intervention arm, these values were 26.8%, 14%, and 23.4%.

The main results of the article on iQiP can be found in Reference 23, where the primary aim was to assess the impact of intervention on short-term abstinence at 8 weeks based on responses from the self-reported questionnaire. All missing responses were set to smoking.^25^ The study reported no statistically significant differences in abstinence at 4 or 8 weeks, but found a significant effect of intervention on long term 6-month abstinence measures. An MNAR SA was also conducted in Reference 23 and suggested that conclusions were largely insensitive to departures from MAR. However, the analysis did not allow data in the two treatments arms to be imputed separately, nor were plausible departures from MAR elicited from expert opinion.

## 3 Missing Data Methods

We are interested in investigating the effect of iQiP intervention on the odds of 2-week abstinence at 8 weeks and 6 months after the quit date, once missing outcome data has been imputed by three different methods: (a) Missing=Smoking; (b) Multiple imputation under MAR; and (c) Multiple imputation under MNAR. These two outcomes are chosen as 8-week abstinence was the primary outcome of interest in Reference 23, and both outcomes were collected by research staff specifically for the research project, whereas the 4-week data were routinely collected by an SCA and have greater potential for reporting bias. Let *Y*_1_ and *Y*_2_ be 8 weeks and 6 months self-reported two-week abstinence from smoking (1 = abstinent and 0 = not abstinent) and let *Z* denote the arm of the trial, where 0 = *Control* (C) and 1 = *Intervention*/*Experimental* (E). The analysis models are

(1) $$\begin{array}{c} logit\left(Pr\left(Y_{1}=1|Z\right)\right)=\alpha_{1}+\beta_{1}Z\\ logit\left(Pr\left(Y_{2}=1|Z\right)\right)=\alpha_{2}+\beta_{2}Z, \end{array}$$

where *β*_1_ and *β*_2_ are the effects of interest. Treatment allocation in the study was randomized, and adjustment for baseline did not affect previous results23 hence the effects of interest are presented as unadjusted odds ratios with 95*%* confidence intervals.

### 3.1 Missing = Smoking

Individuals who do not give their current smoking status are considered more likely to have resumed smoking than those who do respond. The “Russell Standard”25 therefore deals with missing outcome data in smoking cessation trials by setting them all to smoking by default. This is known as the “missing = smoking” assumption. With MNAR imputation, it is to possible to explore the effect of intervention on smoking cessation, while applying less stringent, and possibly more likely penalties to the quit rate of nonresponders.

### 3.2 Multiple imputation under MAR

Multiple imputation involves drawing values for missing data points based on a specified model for the probability distribution of the unobserved data, given the observed data, called the imputation model. Multiple copies of the incomplete data are imputed, and for each copy, the effect(s) of interest is estimated via regression modeling. These estimates and their standard errors are then pooled using Rubin’s rules,^1^ allowing the final analysis to account for the additional uncertainty caused by filling in missing values with imputations. See Reference 26 for further details on multiple imputation. Under the MAR assumption, multiple imputation can be performed using fully conditional specification (FCS) described in Reference 3. In FCS, imputations for missing data are drawn for each *Y_i_* in turn based on a series of univariate imputation models, one for each variable with missing data by regressing on the remaining variables with missing data, as well as any complete variables (typically baseline variables) that are a predictor of missingness.

For this study of iQiP, imputation under MAR will impute each arm separately (as is considered good practice in trial analyses such as Reference 26) under the following imputation models.

(2) $$\begin{array}{c} logit\left(Pr\left(Y_{1}=1|Y_{2},Z=z\right)\right)=\alpha_{1z}+\beta_{1z}Y_{2}\\ logit\left(Pr\left(Y_{2}=1|Y_{1},Z=z\right)\right)=\alpha_{2z}+\beta_{2z}Y_{1} \end{array}$$

for *z* = 0, 1.

Our imputation models include no baseline variables, which were not found to be a significant predictor of missingness at 8 weeks or 6 months. Complete auxiliary variables can be just included as predictors in the imputation models, but those with missing data must also be imputed. For this reason, 4-weeks abstinence was not included in the imputation models (see discussion). The models were fitted by the mice package in R.^27,28^ As the proportion of missing data is on average around 20%, we impute *m* = 20 multiply imputed datasets,^26^ cycling through the variables 10 times (see Section 7).

### 3.3 Multiple imputation under MNAR

Multiple imputation under the MNAR assumption will be performed by the NARFCS procedure of Reference 10. As with FCS, imputations are drawn from a series of univariate models for each variable with missing data, except that these models now also include the missingness indicators of the data. Define *M*_1_ and *M*_2_ as the missingness indicators (1 if missing for that individual and 0 if observed) for *Y*_1_ and *Y*_2_, respectively. If the FCS imputation models are as in Equation (2), the equivalent NARFCS imputation models for missing data in each arm are

(3) $$\begin{array}{l} logit\left(Pr\left(Y_{1}=1|Y_{2},M_{1},M_{2},Z=z\right)\right)={\tilde{\alpha }}_{1z}+{\tilde{\beta }}_{1z}Y_{2}+\gamma_{1z}M_{2}+\delta_{1z}M_{1}\\ logit\left(Pr\left(Y_{2}=1|Y_{1},M_{1},M_{2},Z=z\right)\right)={\tilde{\alpha }}_{2z}+{\tilde{\beta }}_{2z}Y_{1}+\gamma_{2z}M_{1}+\delta_{2z}M_{2}, \end{array}$$

for *z* = 0, 1.

As before we set *m* = 20, cycling through the variables 10 times. Imputation will be performed in R using code developed in Reference 12. Subject to the data being nonmonotone (that is being missing at one follow-up time does not guarantee being missing at all future follow-up times), the $\tilde{\alpha }$, $\tilde{\beta }$, and *γ* terms are estimable from the observed data. The *δ* terms are the SPs of the procedure. These cannot be estimated from the data, but are instead set to specific plausible values by the user before imputation. These can be interpreted as the difference in the log odds ratio of quitting between observed and missing individuals, conditional on *M*_1_, *M*_2_, *Z*, and either *Y*_1_ or *Y*_2_.

Work in Reference 11 notes that eliciting expert opinion on *δ*_1*z*_ and *δ*_2*z*_ may be difficult when they condition on a number of other variables, because this often means asking about nonresponders who are matched in ways that are rarely studied, and thus experts have little knowledge of. The need to elicit on the log odds scale adds further complexity.

To ease the prior elicitation, define $\pi_{1z}^{NR}$ as the *overall quit rate* in missing individuals (that is the proportion of missing individuals who have quit) at 8 weeks in arm *z* and $\pi_{2z}^{NR}$ as the equivalent parameter at 6 months. These are related to the equivalent *δ* parameters in that the log odds scale and conditioning are removed. This gives us related parameters that can be interpreted, and elicited by experts. Our strategy is to elicit $\pi_{1z}^{NR}$ and $\pi_{2z}^{NR}$ and then calculate what values of *δ*_1*z*_ and *δ*_2*z*_ these correspond to. Details are given in Section 5.

## 4 Expert Elicitation

### 4.1 Elicitation aims

The aim of expert elicitation in this study is to obtain, from knowledgeable individuals, prior distributions on their views of the likely values for $\pi_{1z}^{NR}$ and $\pi_{2z}^{NR}$. This involves asking questions about their views of the likely quit rates in nonresponders at 8 weeks and 6 months in both arms, and constructing priors based on their views.

We suspect that an expert’s views about nonresponse on each trial arm will share some similarities. For this reason, we will elicit bivariate prior distributions on $\left(\pi_{1C}^{NR},\pi_{1E}^{NR}\right)$ and $\left(\pi_{2C}^{NR},\pi_{2E}^{NR}\right)$. Rather than asking experts to construct this distribution directly, we elicit marginal prior distributions for $\pi_{1C}^{NR}$ and $\pi_{1E}^{NR}$ and the correlation between their views, and then construct the bivariate prior that corresponds to this information. The correlation represents how much an expert’s beliefs would change about one parameter, if they were given information about the other. This correlation is important, as it will have a major influence on the shape of the bivariate distribution, and thus the plausible regions for the parameters. We will then elicit their views on $\left(\pi_{2C}^{NR},\pi_{2E}^{NR}\right)$.

We will not elicit the correlations of expert opinion between follow-up periods and will also assume that between arm-correlations are the same at both follow-up times. This is intended to simplify and speed up the elicitation process and prevent experts from being discouraged from participating in the elicitation.

### 4.2 Elicitation process

Fifteen experts in the field of smoking cessation were identified based on their experience of conducting smoking cessation trials, and their knowledge of the literature. These experts came from a variety of different institutions and countries and were invited to answer an online elicitation questionnaire, created as an Rshiny package in R studio. The questionnaire is based on that of Reference 9, but adapted for the iQiP analysis. The final draft of this questionnaire can be found at https://mrcbsu.shinyapps.io/iquit9/, and screenshots of the app can be found as Supplementary Material. The questionnaire is linked to a dropbox account, which collects and stores the experts’ responses.

Experts were sent a link to a first draft of the app via email, and given a month to respond. The first app had a text box to provide feedback, and this was used to help create a final version. This final version was sent to the same 15 experts via email and their responses used for the analysis. For the final version, experts were offered to be talked through the app by phone or Skype. Ethical approval for the collection of expert response was given by the UCL Research Ethics Committee, and responses from experts were anonymous. Both the first and final versions received responses from five experts and it is unknown if these were the same five people.

#### Questionnaire App

The first two questions have the same form, and elicit prior distributions on $\pi_{1C}^{NR}$ and $\pi_{1E}^{NR}$, respectively. Experts are given the quit rate in the relevant responders and summary statistics for baseline characteristics of responders and nonresponders. They are then asked to move two sliders to specify parameters of a truncated normal curve over the range (0 − 100)*%*. One slider lets them specify what they think is the most likely quit rate, which is the mode of the distribution. The other slider lets them specify a certainty score, representing how certain they are about their views. This is used to set a value for the standard deviation. Experts can see the curve they are constructing, which reacts dynamically to the sliders to help them answer.

Question 3 elicits the correlation between their views on $\pi_{1C}^{NR}$ and $\pi_{1E}^{NR}$. Experts are asked how much they would change their most likely value of $\pi_{1C}^{NR}$ if they were told the true value of $\pi_{1E}^{NR}$ was at the upper quartile for the curve they gave for $\pi_{1E}^{NR}$. Experts are asked to mark a point on the curve for $\pi_{1C}^{NR}$ representing their updated most likely value, based on this new information for $\pi_{1E}^{NR}$. To help, experts are shown the upper quartile on the curve for $\pi_{1C}^{NR}$, which is the point corresponding to a correlation of 1 between $\pi_{1C}^{NR}$ and $\pi_{1E}^{NR}$.

The app has significant modification to its structure compared to Reference 9, including additional tabs for introductions explaining the questions in detail, baseline characteristics, and additional text explaining some of the mathematical concepts in terms of what they represent about their views. The final two questions are slightly abridged versions of questions 1 and 2, and elicit priors on $\pi_{2C}^{NR}$ and $\pi_{2E}^{NR}$.

Question structure was made more generalizable and, therefore, easier to adapt to other trials than in Reference 9, particularly for question 3. Also included were text boxes asking experts to explain their reasoning and validation tabs at the end of each question. The latter aim to determine how well the question elicited what was intended. These are shown in the screenshots in the Supplementary Material.

### 4.3 Credible regions for $\pi_{1z}^{NR}$ and $\pi_{2z}^{NR}$

Plausible ranges for the *π* parameters are taken by constructing prior credible regions for each of $\left(\pi_{1C}^{NR},\pi_{1E}^{NR}\right)$ and $\left(\pi_{2C}^{NR},\pi_{2E}^{NR}\right)$ using the elicited bivariate priors. These take the form of (1 − *α*)*%* highest density regions (HDRs).29 These are contours over the bivariate density space, typically centered on the mode such that 1 − *α%* of the density lies within the contour. We construct for each expert 50% and 90*%* HDRs, taking the 90*%* HDR as the prior credible region for each expert, with the 50% HDR a visual reference of the distribution.

### 4.4 Opinion pooling

It is typical to use linear opinion pooling methods^13,30^ with expert elicitation with multiple experts. This involves generating a single pooled prior distribution, representing a consensus of expert opinion, from which to make inference about the SPs. A pooled prior distribution of expert opinion can be taken as the mixture distribution of the priors of each expert, and its resultant 90% HDR may then be taken as a pooled prior credible region.

A mixture of truncated bivariate distributions has no simple analytical form. To obtain the HDR, we drew random samples from the pooled prior distribution. As a mixture distribution is simply a weighted sum of the distributions it is mixing, we emulated the drawing of random samples by combining random samples from each expert’s prior. In this way, a linearly pooled HDR can be obtained as follows.

1. Generate *n* random samples from the bivariate prior distribution of each of the *m* experts. Take *n* as a large value (for example, ≥ 100 000).
2. Combine the *m* sets of *n* random samples to form a single set of *mn* samples.
3. Use kernel density estimation (or other relevant method) to estimate the bivariate distribution the *mn* samples are from. This is the pooled bivariate distribution.
4. With the samples in step 2, and density estimates in step 3, construct the 50% and 90% HDRs of the pooled bivariate distribution.

Example code to perform this method in R is given as Supporting Information, and makes use of the “ks” and “hdrcde” packages. With equal numbers of random samples from each expert, the method assumes that all experts provide equal contribution to their pooled consensus. Other pooling methods may be applied by changing the proportion of samples from each expert.

## 5 Sensitivity Analysis

We have elicited plausible ranges for the *π* parameters. However, the imputation models require specification of values for the *δ* parameters. We can link the two sets of parameters using the following SA.

### 5.1 Method

The SA itself has the following basic steps.

1. Choose *s* sets of values for *δ*_1*C*_, *δ*_1*E*_, *δ*_2*C*_, and *δ*_2*E*_. Choice of *s* and the sets of values are described below. For each set of values
2. Impute the missing data using NARFCS.
3. For each imputed dataset calculate *β*_1_ and *β*_2_ from Equation (1) (the effects of interest). Then obtain the pooled estimates and pooled *P*-values for *β*_1_ and *β*_2_.1
4. *Additionally* obtain estimates for $\pi_{1C}^{NR}$, $\pi_{1E}^{NR}$, $\pi_{2C}^{NR}$ and $\pi_{2E}^{NR}$ from the imputed data. For example, to estimate $\pi_{1C}^{NR}$, calculate the proportion of imputed individuals in control who have quit at 8 weeks for each multiply imputed dataset, and take the estimate of $\pi_{1C}^{NR}$ as the average of these values. The remaining *π* estimates can be obtained in an equivalent manner. Once steps 2 to 4 are performed for all *s* sets.
5. Graph the pooled estimates and pooled p values for *β*_1_ against $\left(\pi_{1C}^{NR},\pi_{1E}^{NR}\right)$ via a contour plot, and overlay onto the plot the HDRs for $\left(\pi_{1C}^{NR},\pi_{1E}^{NR}\right)$. Repeat for *β* against $\left(\pi_{2C}^{NR},\pi_{2E}^{NR}\right)$ and draw conclusions.

With this method, it is possible to directly compare *β*_1_ and *β*_2_ against the *π* parameters. Values for the *δ* parameters should be chosen simply to obtain estimates of *π* over the ranges covered by the HDRs, without large distances between estimates.

For step 1, it is infeasible to vary all four *δ* parameters at once, as this will make the number of sets *s* far too large to perform the SA in a reasonable time. We therefore vary the SPs for the control and intervention arms at 8 weeks (*δ*_1*C*_ and *δ*_1*E*_) and set the respective SPs at 6 months (*δ*_2*C*_ and *δ*_2*E*_) to the same values, that is, *δ*_2*z*_ = *δ*_1*z*_. This assumption is discussed in Section 5.2.

We take values for *δ*_1*C*_ and *δ*_1*E*_ in intervals of 0.5 over [−4, 4]. Our *s* sets are then given as every combination of these values, with *δ*_2*C*_ = *δ*_1*C*_ and *δ*_2*E*_ = *δ*_1*E*_. We recommend these ranges as a general rule, but may be widened if necessary.

The R code to perform the analysis is given as Supporting Information and may be used to carry out a similar SA.

### 5.2 Assumptions

The above method varies only two of the four SP in Equation (3), keeping the SA to two dimensions. The method still allows control and intervention nonresponders to behave differently from each other at both follow-up periods. The limitation is that this fixes the correlation of the *π* estimates *between follow-up times*, that is, the correlation between $\pi_{1z}^{NR}$ and $\pi_{2z}^{NR}$. These correlations mainly depend on *δ*_2*z*_ and *δ*_1*z*_, which we have enforced to be equal. We cannot determine what these correlations will be until the SA is performed, but we believe our assumption to normally yield estimates with strong positive correlations between follow-up times. Although expert opinion on between follow-up time correlation was not elicited, we suspect the most plausible situation in this study is that it is highly positive. In Section 7, we discuss this limitation and consider the feasibility of SAs with more than two dimensions and with outcome data on more than two follow-up times.

## 6 Results

### 6.1 Questionnaire app

**Figure 1:**
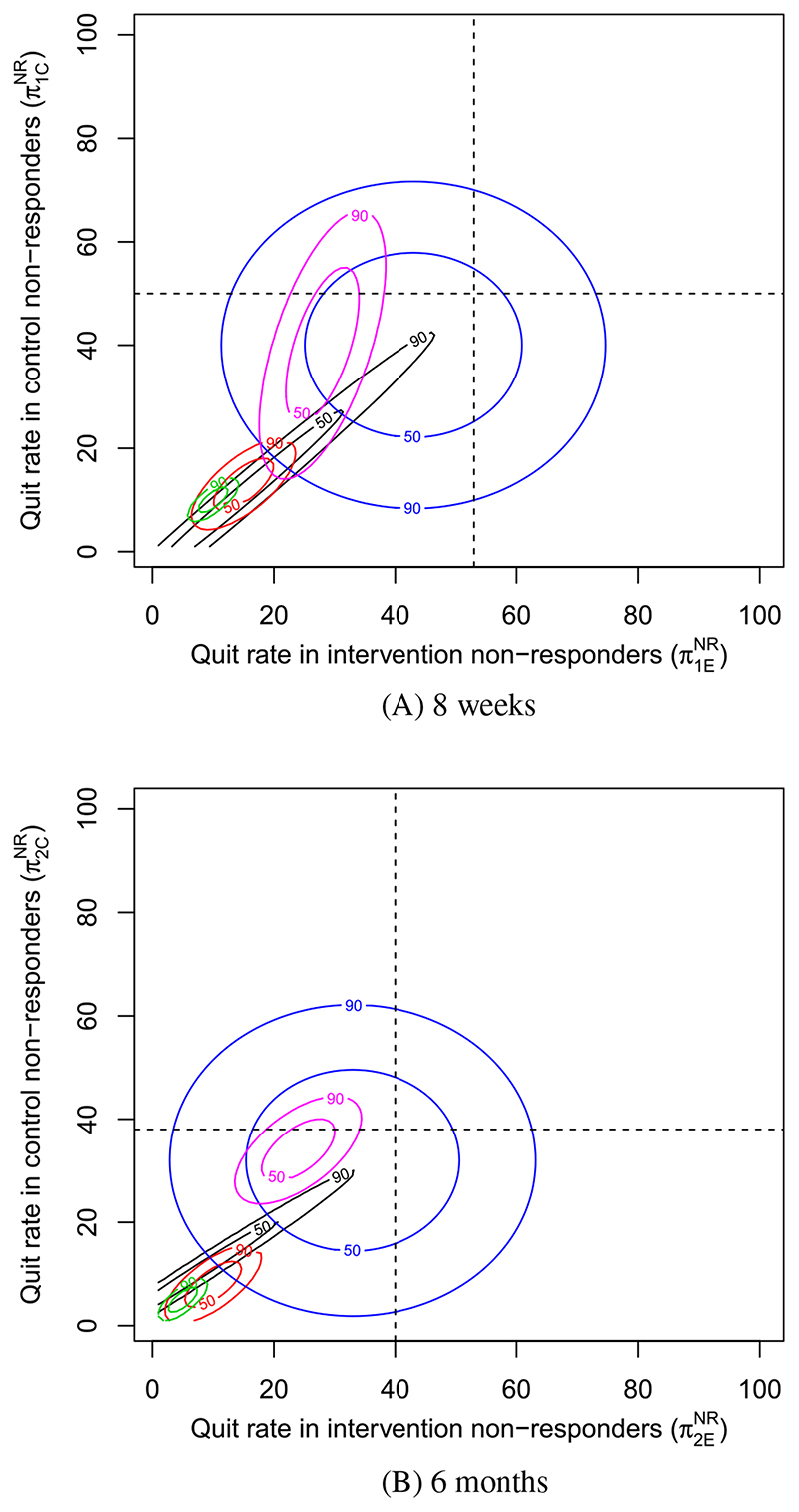
Prior credible regions for smoking cessation in nonresponders for each expert at, A, 8 weeks and, B, 6 months [Colour figure can be viewed at wileyonlinelibrary.com]

The credible regions of each expert at 8 weeks and 6 months are overlaid in Figure 1. Each expert’s region is color coded, and based on their responses to question 3, their derived correlations are as follows: *Black* = 1.0, *Red* = 0.7, *Green* = 0.74, *Blue* = 0.0, *Purple* = 0.6. Note that for the expert in black, a correlation of 0.99 was used to allow construction of the HDR. The observed quit rates in each arm are marked by black dotted lines, with their intersection corresponding to the MAR assumption.

While experts agree that the quit rate in nonresponders is almost surely lower than the quit rate in observed individuals, there is only minor consensus as to how much lower. At 8 weeks, 3 experts (black, red, and green) believe the most likely quit rate for nonresponders in both arms is under 20*%*, close to the typical assumption of missing equals smoking, but the experts in blue and purple believe it to be closer to the observed quit rate in both arms, given as 50% and 53% in the control and intervention arms, respectively. Some consensus can be inferred between experts in black, red, and green, but the credible regions of experts in purple and blue are fairly distinct. The expert in blue is notably different from the rest, giving an elicited correlation of 0, with the other four having given correlations of at least 0.6. As a result, the blue HDRs are the largest credible regions. A similar pattern can be found at 6 months. Experts were in agreement, however, that the quit rate in nonresponders is generally lower at 6 months compared to 8 weeks, likely due to the lower observed quit rates at 6 months.

We note that relative to the observed quit rates, the credible regions at 8 weeks and 6 months are similar, perhaps suggesting that expert opinion between follow-up times is positively correlated.

### 6.2 Credible regions for $\pi_{1z}^{NR}$ and $\pi_{2z}^{NR}$

**Figure 2:**
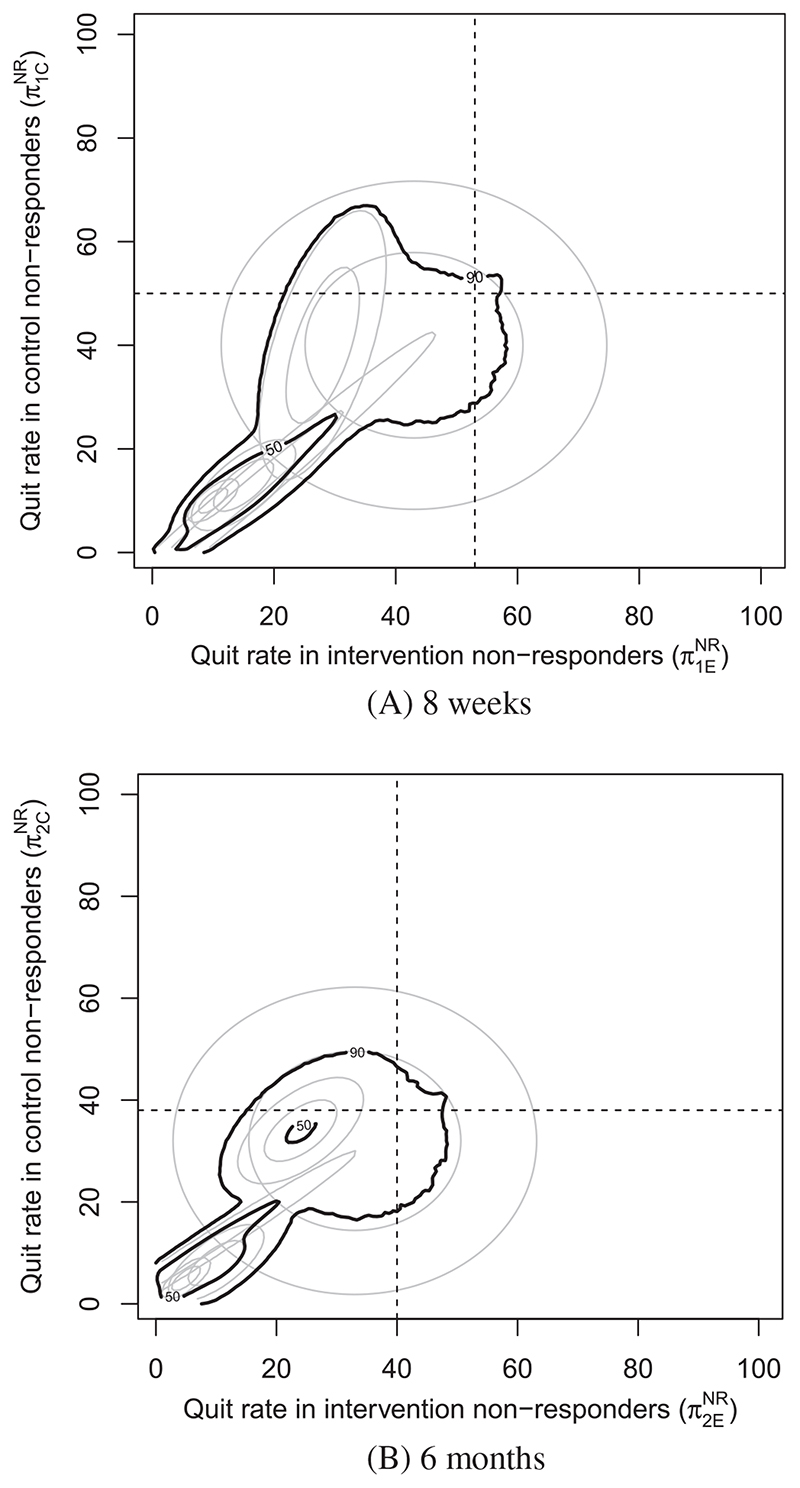
Pooled prior credible regions for smoking cessation at, A, 8 weeks and, B, 6 months

We construct the 50% and 90 *%* pooled HDRs as described in Section 4 in Figure 2. To ensure smooth contours, five million samples from each expert were taken to construct each pooled HDR. The pooled HDRs reinforce the inference from Figures 1 and 2 that credible values for the quit rate in nonresponders are mostly lower than the observed individuals. We note that due to the wide range of opinions expressed by the experts, one could argue that opinion pooling in this instance may not be appropriate. At 8 weeks, the 50 % credible regions of the experts in blue and purple do not intersect the pooled 50% credible region, and the pooled 90% credible regions do not include most values that the blue expert considers 90% credible. As a result, we decided to overlay both the pooled credible regions and the individual credible regions over the SA to draw conclusions.

### 6.3 Sensitivity analysis

Table 1 displays the estimated effect of the intervention on smoking cessation at 8 weeks and 6 months as an odds ratio, when data are imputed under *missing* = *smoking* or MAR assumption. It shows that the effect sizes of intervention vary from 1.05 − 1.22, suggesting a weak improvement in quit rate under intervention. However, the effect of the intervention is not significantly different from 1 in any of the four cases and is consistent with the results found in Reference 23.

**Figure 3:**
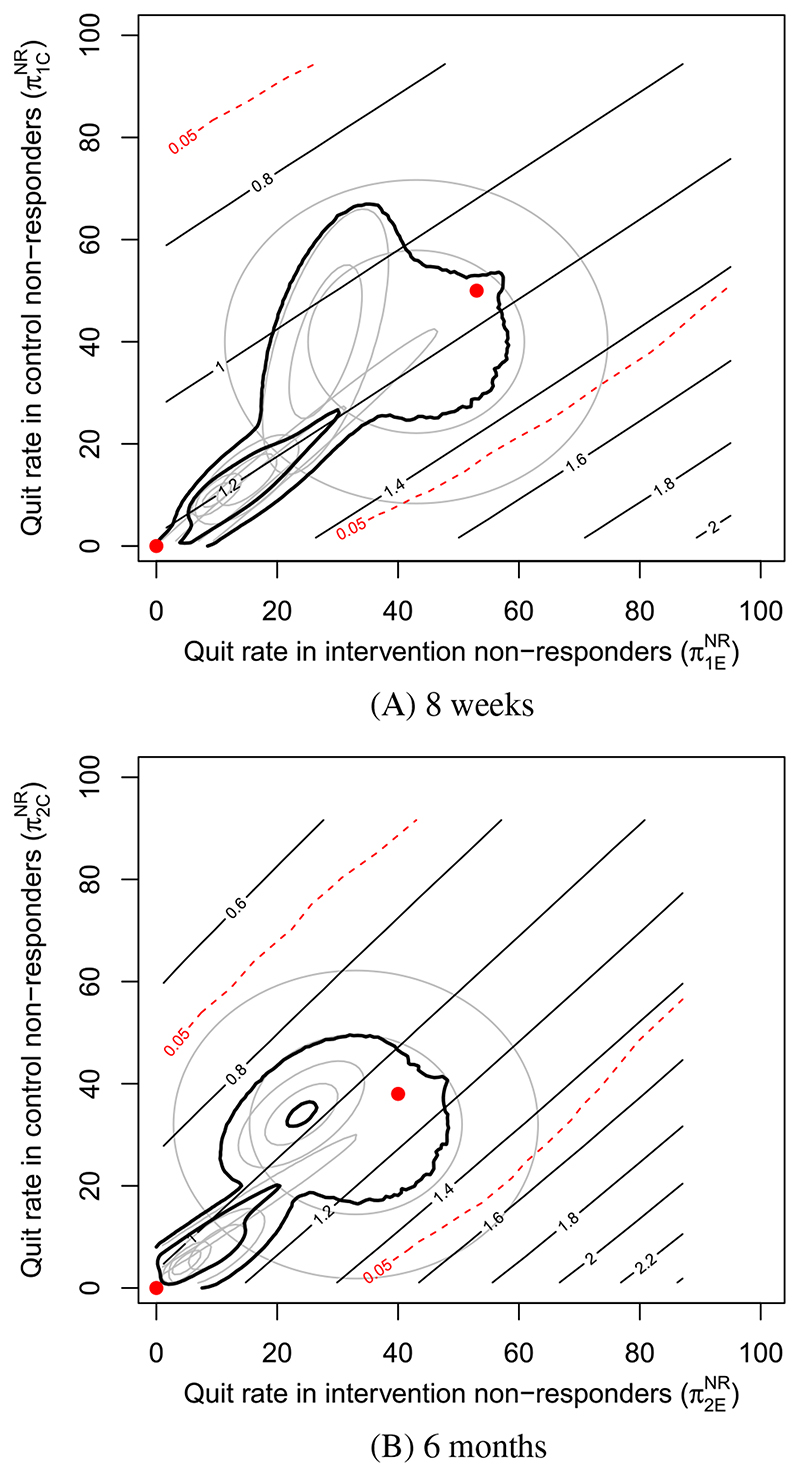
Sensitivity analysis at, A, 8 weeks and, B, 6 months. The straight black contours display effect of intervention (at that time point) on smoking cessation as an odds ratio. The red dashed contours bound the region for which *p* > 0.05. The two red points are placed at (0, 0) (missing equals smoking), and at the quit rates in observed individuals (MAR) [Colour figure can be viewed at wileyonlinelibrary.com]

The MNAR SA described in Section 5 is shown in Figure 3. In Figure 3A, that is, at 8 weeks, the black contours are close to parallel with the identity line (*y* = *x*), meaning that very little change in effect size occurs when the quit rates in nonresponders are similar in both arms. In fact, to observe a notable increase in the intervention effect, the quit rate of nonresponders in intervention has to be quite a lot higher than in control. The red dashed contours bound the region for which the significance test has *p* > 0.05 and indicate that an effect size estimate of around 1.4 to 1.5 is needed to be considered (95%) significant. By observing these red dashed contours, we see that such an effect size estimate requires the quit rate in intervention nonresponders to be around 40% to 50% higher than in control nonresponders. A very similar picture can be seen at 6 months. Such disparities between nonresponder quit rates in each arm are considered highly unlikely by the experts. The pooled credible regions at both follow-up times lie well within the red contours.

The same conclusions can be made from the credible regions of each expert individually, except the expert with the largest HDR. A small section of this expert’s 90% prior credible region does lie outside the red contours, but only at the very edge.

Expert opinion suggests there is little chance that the departures from MAR required to obtain a statistically significant effect of intervention are plausible. The pooled credible regions suggest that the effect size over realistic departures from MAR are around (0.9, 1.3) at each of 8 weeks and 6 months. This is primarily due to the experts in blue and purple, however, as there is little change in the effect size over the credible regions of the other three experts.

Despite the relatively few expert responses and low consensus, the low sensitivity of the effect size of intervention to departures from MAR in these data allows for a conclusion to be made that it is unlikely that intervention performs significantly better than control in improving 8-week and 6-month smoking cessation.

## 7 Discussion and Limitations

The novel contributions of this article include the adaptation of an elicitation app to repeated binary outcomes and the successful use of a novel means of MNAR SA. We also demonstrated the creation of credible regions from expert opinion on two related SPs (in this case for two separate study arms) through the use of highest density regions, including a general means of constructing an HDR representing pooled expert opinion. Our approach has a number of limitations and assumptions, which were all designed to make the elicitation task and the analysis realistic for the experts and the data analyst. We accept that other approaches might be preferable and we discuss some of these limitations below.

### 7.1 Questionnaire app

One limitation is that the app did not elicit correlation at the 6-month follow-up time and assumed it equal to 8 weeks. In the first draft of the app, where correlation was elicited at both follow-up times, experts answered almost identically and the second question was therefore cut to simplify the app.

The results show that the correlation has a significant effect on the size and shape of the prior credible regions and is, therefore, a key piece of information to elicit from expert opinion. It is, however, a major challenge both communicating the concept of prior correlation in nonmathematical terms, and constructing a question that can satisfactorily elicit it. Question 3 was reworked a number of times, but further work on eliciting correlation effectively is needed. Judging by the responses to the validation sections of the app and comments, four of the experts were comfortable with the question, but the expert in blue implied they were not comfortable with the question and gave a correlation of 0, indicating they did not change their view about control nonresponders, given new information about intervention nonresponders. It was decided that this answer constituted a communication of uncertainty, and thus we did not exclude this expert from the analysis. Excluding this expert would not change the conclusions of the MNAR SA, as the resultant pooled HDR would be even narrower. The app elicited expert opinion as truncated normal prior density curves, though this was a simplifying assumption rather than a means to give experts more choice. A simple extension, however, would be to allow experts to choose the truncation points of the normal density curve, allowing them to completely rule out sets of values they believe to be unrealistic. A template app was created to allow users to edit our questionnaire to suit their needs. It is hoped to eventually create a program, which will automatically generate a template based on the number of questions, follow up times, and arms that are required, in which only the images and text boxes need modifying, but is beyond the scope of this article. The template app can be found in the Supporting Information.

A source of disappointment was how few experts responded to the elicitation questionnaire. In Reference 9, the questionnaire was administered while the trial was underway, and experts were invited to answer at a conference, enabling access to a large number of experts who were available and willing to give their opinion. We hoped to show that large numbers of responses could be obtained just by email. However, experience suggests that one needs to identify a lot more than 15 experts, and that they need enthusiastic persuasion and frequent reminders to respond, and even then the response rate would likely be 50*%* or under (Dr Mason, personal communication). Response by email is, therefore viable, but far more experts must be identified, and reminded more frequently.

Due to the lack of consensus in the views of our experts, the pooled HDR may have created a credible region that no expert particularly agrees with. However, a demonstration of its use is important, as the ability to pool opinion with HDRs will be vital for use in future studies. The likely behavior of missing participants remains a very challenging question to pose to experts. We chose experts who were experienced in smoking cessation trials in the belief that they would be best placed to evaluate the nature of participants with missing data, and we provided baseline characteristics of participants with observed and missing data. An improvement would have been to provide detailed information about missing data theory and relevant empirical evidence: we chose not to do this to reduce the burden on our experts. Further work in the area of expert elicitation is of future interest.

### 7.2 Sensitivity analysis

The use of contours of both the substantive effect and significance test allowed for a clear visual picture of the SA. Furthermore, both changes in effect size, and any tipping points (departures from MAR where the significance of conclusions change) may be assessed against credible regions. This gives the method a distinct advantage over most other analysis methods. Furthermore, the results of the SA clearly demonstrate how conclusions can be far more sensitive when the arms of a trial are imputed with different SPs. It is hoped that our methods can allow similar studies with arm specific SPs to be more commonly performed. For example, a similar analysis could have fixed the SPs of the control and intervention arms to be equal, that is, *δ*_1_ = *δ*_1*C*_ = *δ*_1*E*_ and independently vary *δ*_1_ and *δ*_2_. This would investigate sensitivity to departures from MAR when data at each follow up time were allowed to depart from MAR independently, and is a potential question for future work.

Multiple imputation was performed with *m* = 20 multiply imputed datasets. This was based on a common suggestion that *m* should be set to 100 times the fraction of missingness in the missing variables,26 which in this article was approximately 20*%*. Both the MAR analysis and MNAR SA were also run with *m* = 10 and 50 and made very little impact on results. An analyst in practice should set *m* as large as is feasible to obtain the best possible results. Taking values for *δ*_1*C*_ and *δ*_1*E*_ in increments of 0.5 meant that there were 172 = 289 separate sets of values for the SPs for which to run multiple imputation. This increment was chosen as it resulted in acceptably smooth contours for the analysis while also keeping computation time reasonable. One improvement could be to choose values in closer increments such as 0.2 or 0.4. For large datasets, or complicated imputation models with many variables to impute, computation time using this SA could become quite lengthy, and consideration should be given to lowering *m*, reducing the number of sets of values for the SPs, or using cluster computing software.

One improvement to the SA would be to additionally impute and investigate the 4 weeks abstinence measure. This would also allow the imputation models of Equations (2) and (3) to include 4 weeks smoking cessation as a variable, potentially improving imputations. As we suspect, however, that this variable would also be MNAR, this would require additional expert elicitation, and a longer questionnaire. We also would need to be concerned with experts’ views on three separate correlations between follow up times rather than one. This was beyond the scope of the article. However, we detail in the following section an extension to the papers’ SA to allow for more than two follow up times.

#### Extension to more than two follow-up times

One aim of this work is to address how to perform an SA when there are more than two follow-up times. As the number of follow-up times increases, so does the number of SPs in the imputation models, making the SA increasingly difficult. However, the method described in this article can be easily generalized to any number of follow-up times, numbered 1 to *j*, by varying the SPs in the two arms at the first follow-up time, and setting the equivalent SPs in all future follow-up times to the same values as in the first follow-up time, that is, *δ*_1*z*_ = *δ*_2*z*_ = … = *δ*_*jz*_.

This keeps the SA to two dimensions regardless of the number of follow-up times, while still allowing for an in-depth SA at every follow-up time. Ultimately it is felt that any SA that varies more than two SPs independently is likely to be infeasible, as multiple imputation will need to be performed for possibly thousands (or more) sets of values for the SPs. Furthermore, clearly presenting the results of a three or more dimensional SA using contour plots or otherwise is challenging.

The major drawback of this simplification is that the correlations between follow-up times of the elicitable parameters become fixed, and it is possible these correlations may not align with the assumptions of the experts or analysts. Hence, we shall now describe a means to display and validate these correlations, and a means of asserting some control over them. Once the SA in Section 5 is performed, one can either estimate the correlation of the estimates of $\pi_{1z}^{NR}$ and $\pi_{2z}^{NR}$ (or each pair of these estimates when more than two follow-up periods are present), or plot the estimates and infer the correlation.

Based on this evidence, decide if the observed correlations are acceptable. If they are not, re-perform the analysis setting *δ*_1*z*_ = *c*_1_ *δ*_2*z*_ = … = *c*_*j*−1_ *δ*_*jz*_, with *c*_1_, …, *c*_*j*−1_ some constants. General choices for these constants should be informed by the observed graphs and correlations. Future work could consider specific means to calculate these constants based on target correlations, but is beyond the scope of this article. We perform this validation in the Appendix for the paper’s study of iQip, which also compares $\pi_{1z}^{NR}$ and $\pi_{2z}^{NR}$ to the quantiles of expert’s elicited prior density curves, as an alternative validation method.

A simpler alternative may be to impute and analyze each follow-up time separately, but we believe imputations should be informed by the associations between follow-up times.

### 7.3 Instructions to perform an SA

In order to aid in performing similar studies in the future, we provide some instructions as to how to go about an SA with binary data on two follow-up times and two arms.

- Define the analysis model(s) and imputation models based on Equations (1) and (3).
- Create a dropbox account to store responses in. Then open R studio (a separate program to R) and open the “apptemplate.R” file in the Supporting Information containing the template elicitation app. Rename this “app.R” and use it to create your own app, and host it on a server, such as on https://www.shinyapps.io/.
- Identify a list of experts in the relevant area of the study, who would have knowledge about patterns of nonresponse in your trial.
- Send the URL link of this app to your list of experts. Allow for some time to respond, and ideally send periodic reminders.
- Once responses are collected, construct the credible regions using the remaining. R codes provided in the Supporting Information.
- Perform the SA as described in section 5 using the code provided in the Supporting Information and draw conclusions.

## Supplementary Material

Supplementary material

Appendix

## Figures and Tables

**Table 1:** Effect of intervention under the missing equals smoking assumption and MAR imputation as an odds ratio

Missing equals Smoking		
Follow-up time	Estimate	95% CI
8 weeks	1.22	(0.89,1.68)
6 Months	1.05	(0.74,1.49)
**FCS (MAR)**		
**Follow-up time**	**Estimate**	**95% CI**
8 weeks	1.16	(0.83,1.63)
6 Months	1.07	(0.74,1.53)

## Data Availability

The research data of the article is not shared.

## References

[1] Rubin DB (1987). Multiple Imputation for Nonresponse in Surveys.

[2] Carpenter JR, Kenward MG (2013). Multiple Imputation and Its Application.

[3] van Buuren S, Boshuizen HC, Knook DL (1999). Multiple imputation of missing blood pressure covariates in survival analysis. Stat Med.

[4] Kenward M, Goetghebeur E, Molenberghs G (2001). Sensitivity analysis for incomplete categorical data. Stat Modell.

[5] Hollis S (2002). A graphical sensitivity analysis for clinical trials with non-ignorable missing binary outcome. Stat Med.

[6] Diggle P, Kenward MG (1994). Informative drop-out in longitudinal data analysis. J Royal Stat Soc Ser C Appl Stat.

[7] Yan X, Lee S, Li N (2009). Missing data handling methods in medical device clinical trials. J Biopharm Stat.

[8] White IR, Carpenter J, Evans S, Schroter S (2007). Eliciting and using expert opinions about dropout bias in randomized controlled trials. Clin Trials.

[9] Mason A, Gomes M, Grieve R, Ulug P, Powell JT, Carpenter J (2017). Development of a practical approach to expert elicitation for randomised controlled trials with missing health outcomes: application to the IMPROVE trial. Clinical Trials.

[10] Leacy FP (2016). Multiple Imputation Under Missing not at Random Assumptions Via Fully Conditional Specification (PhD thesis).

[11] Tompsett D, Leacy F, Moreno-Betancur M, Heron J, White I (2018). On the use of the not at random fully conditional specification NARFCS procedure in practice. Stat Med.

[12] Moreno-Betancur M, Leacy FP, Tompsett D, White I (2017). Mice: The NARFCS procedure for sensitivity analyses.

[13] O’Hagan A, Buck CE, Daneshkhah A, Eiser R, Garthwaite PH (2006). Uncertain Judgements: Eliciting Expert Probabilities.

[14] Cooke R (1991). Experts in Uncertainty: Opinion and Subjective Probability in Science.

[15] Morgan G, Henrion M (1990). Uncertainty: A Guide to Dealing with Uncertalnty In Quantitative Risk and Policy Analysis.

[16] Colson A, Cooke R (2018). Expert elicitation: using the classical model to validate experts’ judgments. Rev Environ Econ Policy.

[17] O’Hagan T (2013). SHELF: the sheffield elicitation framework.

[18] Oakley J (2019). Tools to support the Sheffield elicitation framework (SHELF) 1.6.0.

[19] Morris D, Oakley J, Crowe J (2014). A web-based tool for eliciting probability distributions from experts. Environ Model Softw.

[20] Ratitch B, O’Kelly M, Tosiello R (2013). Missing data in clinical trials: from clinical assumptions to statistical analysis using pattern mixture models. Pharm Stat.

[21] Leacy F, Floyd S, Yates T, White I (2017). Performing sensitivity analyses to the missing at random assumption using multiple imputation with*δ*-adjustment: application to a tuberculosis/HIV prevalence survey with incomplete HIV status data. Am J Epidem.

[22] Hayati RP, Lee KJ, Simpson JA (2018). Sensitivity analysis within multiple imputation framework using delta-adjustment: application to longitudinal study of Australian children. Longit Life Course Stud.

[23] Naughton F, Jamison J, Boase S (2014). Randomized controlled trial to assess the short-term effectiveness of tailored web-and text-based facilitation of smoking cessation in primary care (iQuit in Practice). Addiction.

[24] Siddiq J, Harel O, Crespi CM, Hedeker D (2014). Binary variable multiple-model multiple imputation to address missing data mechanism uncertainty: Application to a smoking cessation trial. Stat Med.

[25] West R, Hajek P, Stead L, Stapleton J (2005). Outcome criteria in smoking cessation trials: proposal for a common standard. Addiction.

[26] White IR, Wood A, Royston P (2011). Tutorial in biostatistics: multiple imputation using chained equations: issues and guidance for practice. Stat Med.

[27] van Buuren S, Groothuis-Oudshoorn K (2011). Mice: multivariate imputation by chained equations in R. J Stat Softw.

[28] R Core Team (2018). R: A Language and Environment for Statistical Computing.

[29] Hyndman R (1996). Computing and graphing highest density regions. Am Stat.

[30] Genest C, Zidek JV (1986). Combining probability distributions: a critique and an annotated bibliography. Stat Sci.

