# Supplementary material for: A general method for elicitation, imputation, and sensitivity analysis for incomplete repeated binary data
Source: Stat Med. Author manuscript; Available in PMC 2021 Dec 16. (PMC7612109; doi:10.1002/sim.8584)
Supplement: Appendix [file EMS140490-supplement-Appendix.pdf]

SUPPORTING INFORMATION

Additional supporting information may be found online in the Supporting Information section at the end of this article.

How to cite this article: Tompsett D, Sutton S, Seaman SR, White IR. A general method for elicitation, imputation, and sensitivity analysis for incomplete repeated binary data. *Statistics in Medicine*. 2020;39:2921–2935. https://doi.org/10.1002/sim.8584

APPENDIX

The bold black lines in Figure A1A,B below plot the observed estimates of π_{1z}^{NR} and π_{2z}^{NR} in the imputed iQiP data. The results show that in both arms, the observed correlation between the two follow-up times is strongly positive, which is

FIGURE A1 Quantile-quantile plot of the iQiP sensitivity analysis. The bold lines plot the estimates of π_{1z}^{NR} and π_{2z}^{NR} from the sensitivity analysis. The grey lines plot the quantiles of the prior distributions for π_{1z}^{NR} and π_{2z}^{NR} of each expert. A, Control. B, Intervention

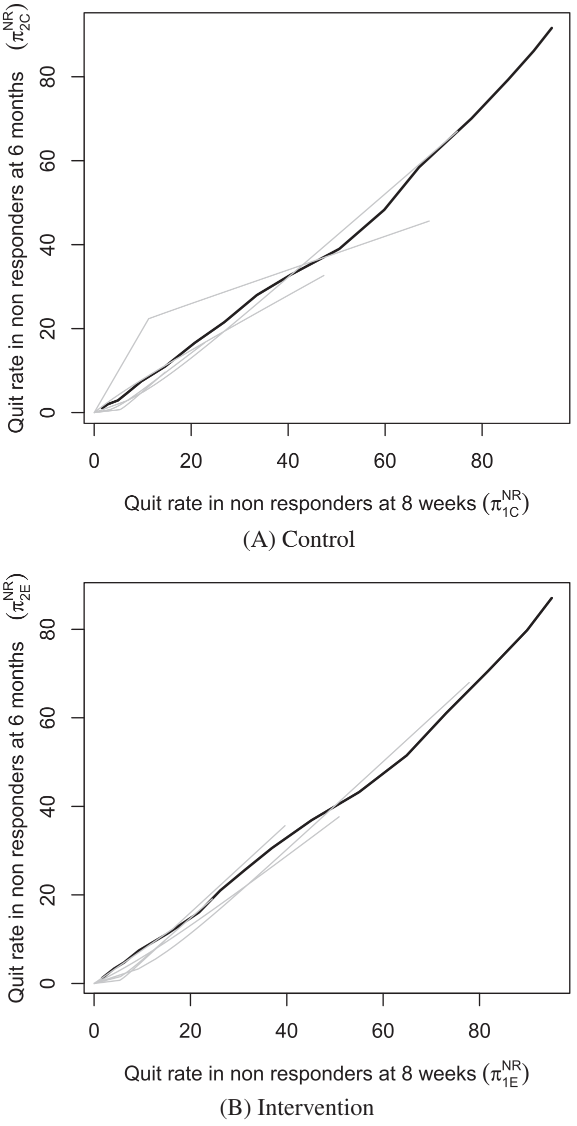

suspected to be the most plausible circumstance in this study. The five grey lines on each figure plot the quantiles of the marginal prior distributions on π_{1z}^{NR} and π_{2z}^{NR} for the five experts. These quantiles lie fairly close to the observed estimates, besides the quantiles for one expert in Figure A1A.
